# BRAF‐V600E immunohistochemistry in a large series of glial and glial–neuronal tumors

**DOI:** 10.1002/brb3.641

**Published:** 2017-02-10

**Authors:** Quentin Breton, Hélène Plouhinec, Delphine Prunier‐Mirebeau, Blandine Boisselier, Sophie Michalak, Philippe Menei, Audrey Rousseau

**Affiliations:** ^1^ Pathology DepartmentAngers University HospitalAngersFrance; ^2^ Genetics and Biochemistry DepartmentAngers University HospitalAngersFrance; ^3^ INSERM UMR‐1066, Micro‐ and Nanomedicine Biomimetics (MINT)Angers University HospitalAngersFrance; ^4^ Neurosurgery DepartmentAngers University HospitalAngersFrance

**Keywords:** allele‐specific quantitative PCR, BRAF‐V600E mutation, E19294, ganglioglioma, glial–neuronal tumor, immunohistochemistry, RRID: AB_11203852, Sanger sequencing, Spring Bioscience VE1

## Abstract

**Introduction:**

Some glial–neuronal tumors (GNT) (pleomorphic xantho‐astrocytoma [PXA], ganglioglioma [GG]) display BRAF‐V600E mutation, which represents a diagnostic clue to these entities. Targeted therapies against BRAF‐V600 protein have shown promising results in GNT. The aim of this study was to assess the utility of BRAF‐V600E immunohistochemistry (IHC, clone VE1) in daily practice in a series of 140 glial, and GNT compared to molecular biology (MB) techniques.

**Methods:**

We performed BRAF‐V600E IHC on all 140 cases. We used Sanger sequencing and allele‐specific quantitative PCR (ASQ‐PCR) to detect BRAF‐V600E mutation when sufficient amount of materiel was available.

**Results:**

BRAF‐V600E immunostaining was detected in 29.5% of cases (41/140 cases; 61.5% GG/GC/AGG (32/52), 33% PXA, 6.6% pilocytic astrocytomas). In 47 cases, MB could be performed: Sanger sequencing and ASQ‐PCR in 34 cases, ASQ‐PCR only in 11 cases, and Sanger sequencing only in two cases. In initial tumors, Sanger sequencing identified BRAF‐V600E mutation in 19.5% tumors (seven of 36 tested cases). ASQ‐PCR showed mutation in 48.5% tumors (17/35 tested cases). In six cases (5 GG, one PXA), the results were discordant between IHC and MB; the five GG cases were immunopositive for BRAF‐V600E but wild type with both MB techniques. In another 7 GG, the percentage of mutated (ganglion) cells was low, and Sanger sequencing failed to detect the mutation, which was detected by IHC and ASQ‐PCR.

**Conclusions:**

In tumors with few mutated cells (e.g., GG), anti‐BRAF‐V600E IHC appears more sensitive than Sanger sequencing. The latter, although considered as the gold standard, is not to be used up‐front to detect BRAF mutation in GG. The combination of IHC and ASQ‐PCR appears more efficient to appraise the indication of targeted therapies in these glioneuronal tumors.

## 1 Introduction

Most primary central nervous system (CNS) neoplasms are gliomas (Dolecek, Propp, Stroup, & Kruchko, 2012); CNS neoplasms with a neuronal component are overall rare (Dolecek et al., 2012). Circumscribed glial and mixed glial–neuronal tumors (GNT) most often develop in children and young adults and are usually low‐grade (grade I or II) according to the 2016 World Health Organization (WHO) classification (Louis et al., 2007).

BRAF is an oncogene mutated in about half of melanomas (BRAF‐V600E mutation in most cases) and in other tumor entities as well (thyroid papillary carcinoma, colorectal carcinoma, hairy cell leukemia, Langerhans cell histiocytosis) (Andrulis, Penzel, Weichert, von Deimling, & Capper, 2012; Garnett & Marais, 2004; Ida et al., 2013; Long et al., 2013; Michaloglou, Vredeveld, Mooi, & Peeper, 2008; Rahman, Salajegheh, Smith, & Lam, 2013; Ritterhouse & Barletta, 2015; Sahm et al., 2012; Schindler et al., 2011). BRAF rearrangements have also been identified in CNS tumors (Brastianos et al., 2014; Dias‐Santagata et al., 2011; Dougherty et al., 2010; Kleinschmidt‐DeMasters, Aisner, Birks, & Foreman, 2013; Koelsche et al., 2013, 2014). Very recently, the mutation V600E has been reported in 96% of papillary craniopharyngiomas (Brastianos et al., 2014). The mutation is also detected in about two‐thirds of pleomorphic xanthoastrocytomas (PXA), one‐third of gangliogliomas (GG) and 20%–25% of dysembryoplastic neuroepithelial tumors (DNT) (Becker et al., 2015; Chappé et al., 2013; Dias‐Santagata et al., 2011; Ichimura, Nishikawa, & Matsutani, 2012; Jones et al., 2008; Koelsche et al., 2013; Rodriguez, Lim, Bowers, & Eberhart, 2013; Roth et al., 2015). Moreover, pilocytic astrocytoma (PA) is characterized by a fusion between the BRAF gene and the locus KIAA1549 (chromosome 7q34) (Faulkner et al., 2015; Jones et al., 2008; Roth et al., 2015). The fusion causes a constitutional activation of the tyrosine kinase domain of BRAF and a permanent activation of the MAP kinase pathway (MAPK) (Roth et al., 2015). Of interest, cerebellar PA harbor the BRAF fusion in about 80% of cases while supratentorial (hemispheric) PA present with the fusion in 29% of cases and with the BRAF‐V600E mutation in about 5% of cases (Becker et al., 2015; Faulkner et al., 2015; Ichimura et al., 2012; Jones et al., 2008; Rodriguez et al., 2013). Distinguishing those circumscribed GNT, characterized by an overall favorable prognosis, from diffuse gliomas (diffuse astrocytomas, oligodendrogliomas), that present a dismal prognosis, may be difficult by histopathology alone. The detection of a BRAF rearrangement has first diagnostic implications as diffuse gliomas do not usually display such an anomaly. Second, it has therapeutic implications as targeted therapies against mutated BRAF‐V600 protein have been recently developed (vemurafenib, dabrafenib) (Hertzman Johansson & Egyhazi Brage, 2014; Lee, Ruland, LeBoeuf, Wen, & Santagata, 2014; Peters et al., 2014; Rutkowski & Blank, 2014). In routine settings, BRAF‐V600E mutation can be identified using Sanger sequencing or allele‐specific quantitative polymerase chain reaction (ASQ‐PCR) (Ihle et al., 2014). Molecular biology (MB) techniques are expensive and not yet widely available. A BRAF‐V600E antibody has recently been commercialized (Capper et al., 2011; Colomba et al., 2013; Ritterhouse & Barletta, 2015), and it is sensitive and specific in detecting BRAF‐V600E mutation in cutaneous melanomas (Capper et al., 2011; Colomba et al., 2013; Long et al., 2013). In contrast, few studies have assessed the reliability of anti‐BRAF‐V600E immunohistochemistry (IHC) in CNS tumors (Behling et al., 2016; Chappé et al., 2013; Long et al., 2013)**.** Some groups have found that BRAF‐VE1 IHC was suboptimal in characterizing brain tumor tissue and that MB techniques are required for a reliable clinical assessment. The aim of this study was to assess the utility of BRAF‐V600E IHC compared to MB on a large series of glial and GNT.

## 2 Materials and methods

One hundred and forty formalin‐fixed paraffin‐embedded samples obtained from 140 patients were retrieved from the archives of the Pathology Department of Angers University Hospital. Thirty‐five recurring tumors were also obtained from the same cohort of patients. The samples were obtained through biopsy or surgical excision between December 1993 and January 2014. Tumors with the following histopathological diagnoses were selected: PA, pilomyxoid astrocytoma (PMA), ganglioglioma/gangliocytoma (GG/GC), anaplastic ganglioglioma (AGG), pleomorphic xanthoastrocytoma (PXA), anaplastic pleomorphic xanthoastrocytoma (APXA), dysembryoplastic neuroepithelial tumor (DNT), desmoplastic infantile ganglioglioma (DIG), astroblastoma (AB), and papillary glioneuronal tumor (PGNT). All cases were reviewed by two pathologists (one junior [QB] and one expert neuropathologist [AR]) and classified according to the 2016 WHO classification of tumors of the CNS (Louis et al., 2007). The entire cohort is described in Table 1. For 131 of 140 patients, tumor material from the initial surgery (biopsy or resection) was available. For nine patients (cases no. 11, 14, 27, 41, 68, 114, 133, and 140; see Table 1), the initial sample was not available (exhausted material, surgery at an outside institution), and only the sample obtained at recurrence was reviewed. The protocol and procedures employed were reviewed and approved by the appropriate institutional review committee. The patients were first treated in the Department of Neurosurgery of Angers University Hospital and those who required adjuvant treatment were followed in the pediatric Oncology Department or at the Western Cancer Institute of Angers (Institut de Cancérologie de l'Ouest [ICO]).

**Table 1:** Cohort of 140 patients

Case no.	Sex	Location	Side	Diag	IHC	Sanger	ASQ‐PCR
1	F	Cerebral hemispheres	Left	AB	+	N/A	N/A
2	M	Cerebellum	Right	PA	−	N/A	N/A
3	F	Basal ganglia	Right	PA	−	N/A	N/A
4	F	Brainstem	Midline	PA	−	N/A	N/A
5	M	Brainstem	Right	PA	−	WT	WT
6	F	Basal ganglia	Right	PA	+	N/A	V600
7	M	Optic tracts	Left	PA	−	N/A	N/A
8	F	Cerebellum	Vermis	PA	−	N/A	N/A
9	M	Optic tracts	Midline	PA	−	N/A	N/A
10	M	Basal ganglia	Left	PA	−	WT	WT
11	M	Cerebral hemispheres	Left	PA	−	WT	N/W
12	F	Basal ganglia	Left	PA	−	N/A	N/A
13	F	Cerebellum	Left	PA	−	N/A	N/A
14	F	Cerebral hemispheres	Midline	PA	−	N/A	N/A
15	M	Optic tracts	Midline	PA	−	N/A	N/A
16	F	Optic tracts	Midline	PA	−	N/A	N/A
17	F	Cerebellum	Vermis	PA	−	N/A	N/A
18	F	Third ventricle	Left	PA	−	N/A	N/A
19	F	Cerebellum	Right	PA	−	N/A	N/A
20	M	Cerebral hemispheres	Left	PA	−	N/A	N/A
21	F	Optic tracts	Midline	PA	−	N/A	N/A
22	M	Cerebellum	Vermis	PA	−	N/A	N/A
23	F	Third ventricle	Midline	PA	−	N/A	N/A
24	F	Basal ganglia	Right	PA	−	N/A	N/A
25	F	Optic tracts	Right	PA	−	N/W	N/W
26	M	Third ventricle	Midline	PA	+	N/A	N/A
27	F	Cerebral hemispheres	Left	PA	−	WT	WT
28	M	Basal ganglia	Left	PA	−	N/A	N/A
29	M	Brainstem	Midline	PA	−	N/A	N/A
30	M	Optic tracts	Right	PA	−	N/A	N/A
31	M	Cerebellum	Right	PA	−	WT	WT
32	F	Optic tracts	Midline	PA	−	N/A	N/A
33	M	Cerebral hemispheres	Right	PA	−	N/A	N/A
34	F	Cerebellum	Vermis	PA	−	WT	WT
35	F	Cerebellum	Vermis	PA	−	N/A	N/A
36	M	Optic tracts	Midline	PA	−	N/A	N/A
37	F	Cerebellum	Right	PA	−	N/A	N/A
38	F	Optic tracts	Midline	PA	−	N/A	N/A
39	M	Cerebral hemispheres	Right	PA	−	N/A	N/A
40	F	Optic tracts	Midline	PA	+	N/A	N/A
41	F	Brainstem	Midline	PA	−	N/A	N/A
42	F	Spinal cord	Midline	PA	−	N/A	N/A
43	F	Cerebellum	Vermis	PA	−	N/A	N/A
44	M	Basal ganglia	Midline	PA	−	N/A	N/A
45	F	Cerebellum	Vermis	PA	−	N/A	N/A
46	F	Optic tracts	Midline	PA	+	WT	V600
47	F	Cerebellum	Right	PA	−	N/A	N/A
48	F	Pineal region	Midline	PA	−	N/A	N/A
49	F	Optic tracts	Midline	PA	−	N/A	N/A
50	F	Third ventricle	Midline	PA	−	WT	WT
51	F	Cerebellum	Right	PA	−	WT	WT
52	M	Cerebellum	Right	PA	−	N/A	N/A
53	F	Cerebellum	Left	PA	−	N/A	N/A
54	F	Cerebral hemispheres	Left	PA	−	N/A	N/A
55	F	Optic tracts	Midline	PA	−	N/A	N/A
56	M	Cerebral hemispheres	Right	PA	−	WT	WT
57	F	Basal ganglia	Right	PA	−	WT	WT
58	F	Brainstem	Midline	PA	−	N/A	N/A
59	F	Cerebellum	Right	PA	−	N/A	N/A
60	M	Basal ganglia	Right	LGGNT	−	N/A	N/A
61	F	Optic tracts	Midline	PMA	−	N/A	N/A
62	M	Optic tracts	Midline	PMA	−	N/A	N/A
63	F	Cerebral hemispheres	Left	DNT	−	N/A	WT
64	M	Cerebral hemispheres	Right	DNT	−	N/A	N/A
65	F	Cerebral hemispheres	Left	DNT	−	N/A	WT
66	M	Cerebral hemispheres	Right	DNT	−	N/A	WT
67	M	Cerebral hemispheres	Left	DNT	−	N/A	N/A
68	F	Cerebral hemispheres	Right	DNT	−	N/A	N/A
69	F	Cerebral hemispheres	Right	DNT	−	N/A	WT
70	M	Cerebral hemispheres	Left	GC	+	WT	V600
71	F	Cerebral hemispheres	Right	GC	−	N/A	N/A
72A^a^	M	Spinal cord	Midline	GC	+ GC	WT	WT
72B^a^	M	Cerebellum	Multiple	GG	+ GC	N/A	N/A
73	M	Cerebral hemispheres	Left	GG	−	WT	WT
74	F	Optic tracts	Midline	GG	+ GC	WT	V600
75	M	Cerebral hemispheres	Right	GG	−	N/A	N/A
76	M	Cerebral hemispheres	Left	GG	−	N/A	N/A
77	F	Cerebellum	Vermis	GG	−	WT	WT
78	M	Optic tracts	Midline	GG	Diff +	N/A	N/A
79	F	Cerebral hemispheres	Left	GG	Diff +	N/A	V600
80	M	Cerebellum	Left	GG	Diff +	N/A	N/A
81	M	Cerebral hemispheres	Left	GG	+ GC	N/A	V600
82	M	Cerebellum	Vermis	GG	Diff +	N/A	N/A
83	F	Optic tracts	Midline	GG	Diff +	N/A	N/A
84	M	Cerebral hemispheres	Left	GG	Diff +	N/A	N/W
85	F	Cerebral hemispheres	Left	GG	−	N/A	N/A
86	F	Cerebellum	Vermis	GG	Diff +	V600E	V600
87	M	Cerebral hemispheres	Right	GG	+ GC	WT	WT
88	F	Optic tracts	Midline	GG	Diff +	N/A	N/A
89	F	Cerebral hemispheres	Right	GG	+ GC	WT	V600
90	M	Cerebellum	Left	GG	−	N/A	N/A
91	M	Optic tracts	Midline	GG	Diff +	V600E	V600
92	M	Cerebral hemispheres	Left	GG	+ GC	N/A	V600
93	M	Cerebral hemispheres	Left	GG	−	N/A	N/A
94	F	Cerebellum	Left	GG	−	N/A	N/A
95	M	Cerebral hemispheres	Left	GG	−	WT	WT
96	F	Cerebral hemispheres	Right	GG	+ GC	WT	V600
97	M	Cerebral hemispheres	Right	GG	Diff +	N/A	WT
98	M	Cerebral hemispheres	Left	GG	Diff +	N/A	N/W
99	F	Cerebellum	Right	GG	+ GC	N/A	N/A
100	M	Cerebellum	Vermis	GG	+ GC	WT	V600
101	M	Cerebral hemispheres	Left	GG	−	WT	WT
102	M	Cerebral hemispheres	Right	GG	Diff +	N/A	WT
103	M	Cerebellum	Vermis	GG	Diff +	WT	V600
104	F	Optic tracts	Right	GG	Eq	N/A	N/A
105	F	Brainstem	Left	GG	Diff +	N/A	N/A
106	M	Cerebral hemispheres	Left	GG	−	N/A	N/A
107	F	Cerebellum	Vermis	GG	+ GC	N/A	N/A
108	M	Spinal cord	Midline	GG	−	N/A	N/A
109	M	Cerebral hemispheres	Right	GG	−	N/A	N/A
110	M	Cerebral hemispheres	Left	GG	+ GC	V600E	V600
111	M	Cerebral hemispheres	Right	GG	Diff +	WT	V600
112	M	Cerebral hemispheres	Left	GG	−	N/A	N/A
113	M	Cerebral hemispheres	Right	GG	Diff +	N/A	N/W
114	M	Cerebral hemispheres	Left	GG	−	N/A	N/A
115	F	Cerebral hemispheres	Left	GG	Eq	WT	N/W
116	M	Cerebral hemispheres	Right	GG	Diff +	N/A	N/W
117	M	Cerebral hemispheres	Right	GG	Diff +	N/A	N/A
118	F	Optic tracts	Midline	GG	Diff +	N/A	N/A
119	M	Cerebellum	Left	GG	−	N/A	N/A
120	M	Cerebellum	Left	LGGNT	−	N/A	N/A
121	M	Cerebellum	Vermis	LGGNT	−	N/A	N/A
122	M	Basal ganglia	Left	LGGNT	−	N/A	N/A
123	F	Cerebral hemispheres	Left	LGGNT	−	N/A	N/A
124	F	Basal ganglia	Right	AGG	−	WT	WT
125	M	Cerebral hemispheres	Right	AGG	+ GC	WT	WT
126	F	Cerebral hemispheres	Left	DIG	−	N/A	N/A
127	F	Cerebral hemispheres	Right	LGGNT	−	N/A	N/A
128	F	Cerebral hemispheres	Left	LGGNT	+	V600E	V600
129	M	Spinal cord	Midline	LGGNT	Eq	N/A	N/A
130	F	Cerebral hemispheres	Right	LGGNT	−	N/A	N/A
131	M	Cerebral hemispheres	Left	PGNT	−	N/A	N/A
132	F	Cerebral hemispheres	Left	PXA	+	N/A	V600
133	F	Cerebral hemispheres	Right	PXA	−	N/A	N/A
134	M	Cerebral hemispheres	Right	PXA	+	V600E	V600
135	F	Cerebral hemispheres	Left	PXA	−	N/A	N/A
136	M	Cerebral hemispheres	Right	PXA	−	N/A	N/A
137	F	Cerebral hemispheres	Left	PXA	+	V600E	V600
138	F	Cerebral hemispheres	Left	APXA	−	WT	N/A
139	M	Cerebral hemispheres	Left	APXA	−	N/A	N/A
140	M	Cerebellum	Right	APXA	−	WT	WT

F, female; M, male; Diag, histopathological diagnosis; IHC, immunohistochemistry; AB, astroblastoma; PA, pilocytic astrocytoma; PMA, pilomyxoïd astrocytoma; DNT, dysembryoplastic neuroepithelial tumor; GC, gangliocytoma; GG, ganglioglioma; AGG, anaplastic ganglioglioma; DIG, desmoplastic infantile ganglioglioma; LGGNT, low‐grade glioneuronal tumor (with varying features); PGNT, papillary glioneuronal tumor; PXA, pleomorphic xantho‐astrocytoma; APXA, anaplastic pleomorphic xantho‐astrocytoma; +, positive; −, negative; + GC, positive ganglion cells; Diff +, diffusely positive; Eq, equivocal; ASQ‐PCR, allele‐specific quantitative PCR; N/A, not assessed; N/W, not workable; V600E, presence of BRAF‐V600E mutation; WT, wild type.

a Cases no. 72A and 72B correspond to synchronous tumors from the same patient.

### 2.1 Immunohistochemical study

Anti‐BRAF‐V600E IHC (mouse monoclonal antibody, VE1 clone, E19294, RRID: AB_11203852, Spring Bioscience, Pleasanton, CA, USA) was performed on 4‐μm‐thick slides on all 175 cases (140 initial samples and 35 recurring tumors). The slides were placed in a Leica BOND III PLC^®^ (Leica Biosystem, Newcastle, UK) and were subjected to antigen retrieval using buffer ER2 (Epitope Retrieval 2) for 20 min. The incubation time with the primary antibody (1/100 dilution) was 20 min. The developing system was the kit Bond Polymer Refine Detection DS9800^®^ (Leica Biosystems, Newcastle, UK). Anti‐BRAF‐V600E immunostaining was considered positive if cytoplasmic staining of tumor cells, either ganglion cells and/or glial cells, was observed. The immunolabeling was considered negative in the absence of staining or in the case of nuclear staining with no cytoplasmic staining or in the case of staining of non tumor cells (inflammatory cells, endothelial cells). When it was not possible to distinguish a weak immunostaining from a background staining, the case was considered “equivocal”. For GNT, immunostaining of glial and neuronal components was evaluated separately. In some cases, additional IHC was performed, using antibodies against the following antigens: synaptophysin (mouse monoclonal antibody, clone Snp88, BioGenex, Netherlands), CD34 (mouse monoclonal antibody, QBEnd‐10 clone, Dako, Denmark), IDH1‐R132H (mouse monoclonal antibody, clone H09, Dianova, Germany), GFAP (mouse monoclonal antibody, clone 6F2, Dako, Denmark), Olig‐2 (goat monoclonal antibody, AF2418, R&D Systems, Denmark), neurofilament (mouse monoclonal antibody, 2F11 clone, Monosan, Netherlands), chromogranin A (mouse monoclonal antibody, DAK‐A3 clone, Dako, Denmark), internexin‐alpha (mouse monoclonal antibody, clone 2E3, Novus Biologicals, UK), and p53 (mouse monoclonal antibody, clone DO‐7, Dako, Denmark) (results not shown).

### 2.2 Detection of BRAF‐V600E mutation by molecular biology

Detection of BRAF‐V600E mutation was performed by MB on 53 samples (51 initial tumors and two recurrences) in the Department of Genetics and Biochemistry of Angers University Hospital (DPM). Contributing results were obtained only in 47 of 53 samples. Briefly, five 10 μm‐thick slides were used for DNA extraction. DNA was extracted and purified using the Nucleospin FFPE DNA kit^®^ (Macherey‐Nagel, Düren, Germany) according to the manufacturer's instructions. BRAF‐V600E status was assessed by allele‐specific quantitative PCR (ASQ‐PCR) and direct sequencing (Sanger) in 34 cases, ASQ‐PCR alone in 11 cases, and direct sequencing alone in two cases. The Sanger sequencing was performed with 10 μl of purified DNA using the sequencer CEQ 8000 or 8800 Genetic Analysis System^™^ (Beckman Coulter, Fullerton, CA, USA). ASQ‐PCR was performed using a Chromo 4 Real‐Time PCR detector (Bio‐Rad, Hercules, CA, USA) or a FAST unit 7500 (Applied Biosystems, Foster City, CA, USA). A first ASQ‐PCR was performed with primers BRAF‐MASA‐WT‐F (sequence GTGATTTTGGTCTAGCTACAGA) and BRAF‐MASA‐R (sequence GGCCAAAAATTTAATCAGTGGA). In parallel, an ASQ‐PCR with primers BRAF‐MASA‐MUT‐F (sequence GTGATTTTGGTCTAGCTACAGT) and BRAF‐MASA‐R (sequence GGCCAAAAATTTAATCAGTGGA) was carried out. The primers were obtained from Eurogentec (Liège Science Park, Liège, Belgium). The molecular biologist interpreting the sequencing data was blinded to the anti‐BRAF IHC results. All cases with discordant BRAF status in IHC versus Sanger sequencing were tested by the two techniques (Sanger sequencing and ASQ‐PCR).

### 2.3 Statistical analysis

IHC results (positive, negative, or equivocal) were compared with histopathological diagnosis, tumor location, and age at diagnosis (pediatric [<18 years] versus adult [≥18 years]) using Fisher's exact test. A *p* value <.05 was considered significant.

## 3 Results

### 3.1 Clinical and radiological data

One hundred and forty patients were included. The mean age at initial diagnosis was 16.2 years (standard deviation 14 years, range 7 months to 74 years). There were 68 males and 72 females (sex ratio M/F: 0.94). The tumor was located in the cerebellum in 32 cases, in the opto‐chiasmatic region in 23 cases, in the cerebral hemispheres in 60 cases, in the basal ganglia in 11 cases, in the brainstem in six cases, in the region of the third ventricle in four cases, in the spinal cord in four cases, and in the pineal region in one case. The entire cohort is described in Table 1.

### 3.2 Histopathology

**Figure 1:**
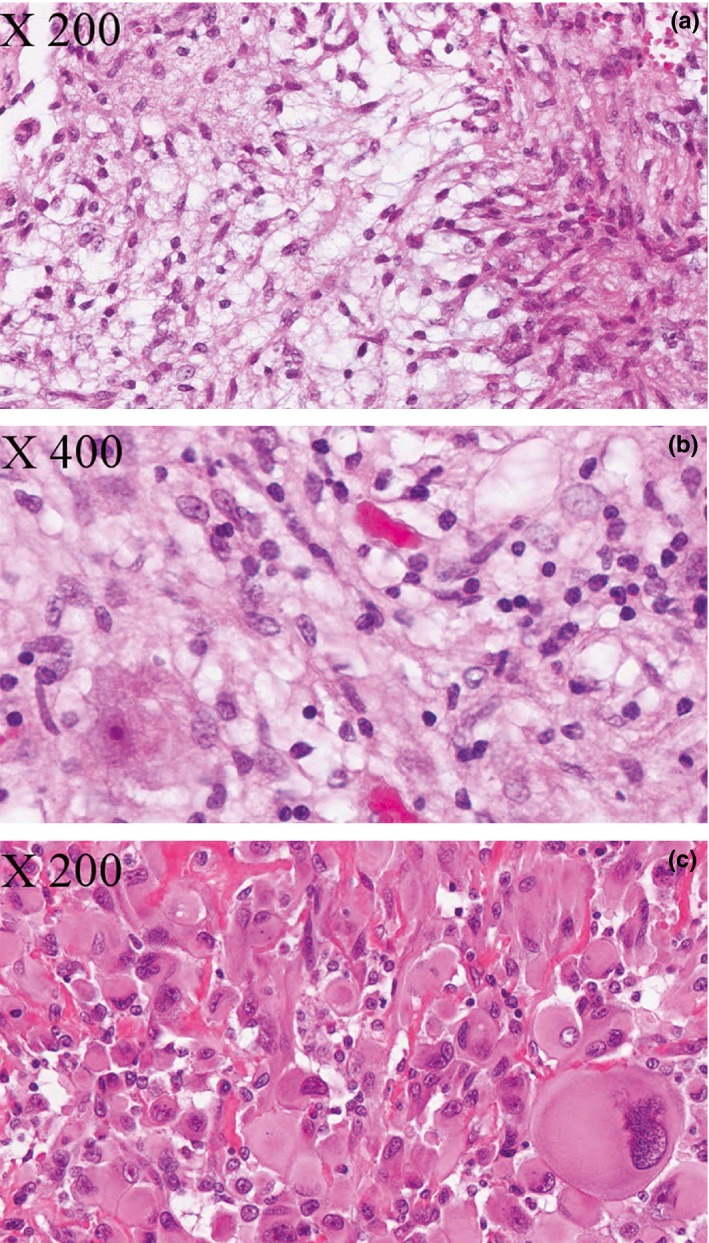
Histopathological aspects of glial and glioneuronal tumors. (a) Pilocytic astrocytoma (PA) (case no. 10). Glial tumor composed of elongated bipolar cells; (b) ganglioglioma (case no. 102). Tumor with a glial component similar to that of PA intermixed with mature neurons (ganglion cell, bottom left); (c) pleomorphic xantho‐astrocytoma (case no. 134). Glial tumor with large pleomorphic cells, often atypical or “bizarre‐looking” (bottom right). Hematoxylin Phloxine Saffron(HPS)‐stained slides

The histopathological diagnosis for the 140 patients was as follows: PA (58 cases), PMA (two cases), GG/GC (50 cases), AGG (two cases), DNT (seven cases), PXA (six cases), APXA (three cases), astroblastoma (one case), DIG (one case), and PGNT (one case) (Table 1). Nine cases of low‐grade GNT with varying features suggestive of PA, GG, or DNT were also included (hereafter referred to as LGGNT). Representative microscopic features are shown in Figure 1.

### 3.3 Immunohistochemical study

**Figure 2:**
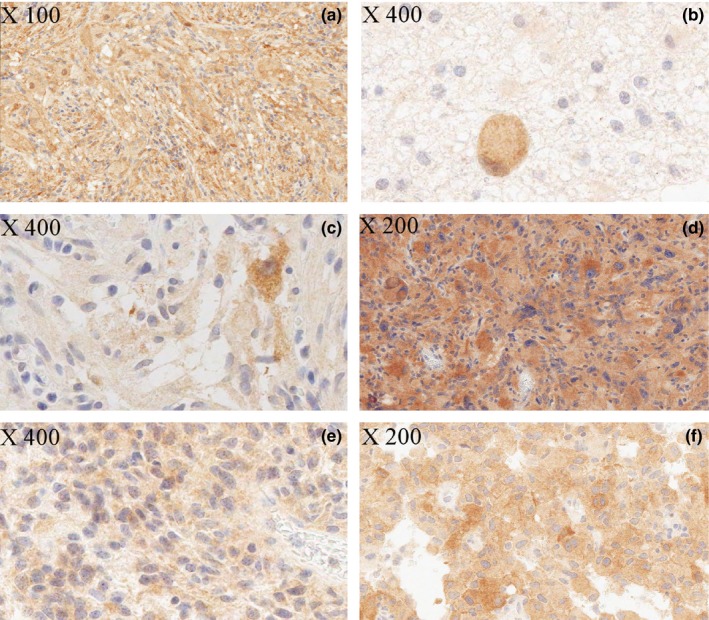
BRAF‐V600E immunostaining. (a) Ganglioglioma (case no. 102) with immunostaining of both tumor components, glial and neuronal. (b) Anaplastic ganglioglioma (case no. 125) with moderate immunostaining of the neuronal component. (c) Ganglioglioma (case no. 118) with diffuse (glial and neuronal) immunostaining but with a more intense staining in the neuronal component. (d) Pleomorphic xantho‐astrocytoma (case no. 132) with diffuse intense immunostaining of the tumor cells. (e) Pilocytic astrocytoma of the basal ganglia (case no. 6) with diffuse staining of the tumor cells. (f) Astroblastoma (case no. 1) with diffuse staining of the tumor cells

Anti‐BRAF‐V600E IHC was performed on all samples (140 initial tumors and 35 recurring tumors). Immunoreactivity with BRAF‐V600E antibody was detected in 41 of 140 patients (29.5%). Immunopositivity was observed in 31 of 50 GG/GC (62%), 1 of 2 AGG (50%), 3 of 6 PXA (50%), and 0 of 3 APXA. Only 4 of 60 PA/PMA (6.6%) expressed BRAF‐V600E; the two PMA cases were immunonegative. The astroblastoma and one LGGNT (case no. 128) were immunopositive (Figure 2). The seven DNT, the DIG, and the PGNT did not express BRAF‐V600E. The results of anti‐BRAF IHC according to tumor location and histopathological diagnosis are presented in Tables 1 and 2.

**Table 2:** Results of anti‐BRAF immunohistochemistry according to histopathology

Histopathology	Positive IHC	Negative IHC	Equivocal cases	Total
AB	1 (100%)	0		1
PA/PMA	4 (6.6%)	56 (93.4%)		60
DNT	0	7 (100%)		7
GG	32 (63%)	17 (34%)	2 (4%)	50
AGG	1 (50%)	1 (50%)		2
PXA	3 (50%)	2 (33%)	1 (17%)	6
APXA	0	3 (100%)		3
DIG	0	1 (100%)		1
PGNT	0	1 (100%)		1
LGGNT	1 (11%)	7 (78%)	1 (11%)	9

IHC, immunohistochemistry; AB, astroblastoma; PA, pilocytic astrocytoma; PMA, pilomyxoid astrocytoma; DNT, dysembryoplastic neuroepithelial tumor; GG, ganglioglioma; AGG, anaplastic ganglioglioma; PGNT, papillary glioneuronal tumor; PXA, pleomorphic xantho‐astrocytoma; APXA, anaplastic pleomorphic xantho‐astrocytoma; DIG, desmoplastic infantile ganglioglioma; PGNT, papillary glioneuronal tumor; LGGNT, low‐grade glioneuronal tumor (with varying features).

For GG/GC/AGG, BRAF‐V600E protein expression was detected in 32 of 52 patients (61.5%). Out of all GG/GC/AGG tested, 61.5% had diffuse cytoplasmic staining in both glial and ganglion cell components while 38.5% displayed cytoplasmic staining only in the ganglion cells (Figure 2). Interestingly, no case had an immunostaining restricted to the glial component. There was often a slight background staining, sometimes difficult to distinguish from a weak genuine immunostaining. Two cases (no. 104 and no. 115) were considered equivocal. There was not enough material left to perform MB on case no. 104. Case no. 115 showed no BRAF mutation by Sanger sequencing and ASQ‐PCR failed.

Three PXA (3/6 cases, 50%) were immunopositive, and all three APXA (3/3, 100%) were immunonegative. The three immunopositive PXA displayed diffuse staining with high (two cases) to moderate (one case) intensity. Another case of PXA was equivocal, showing a weak diffuse cytoplasmic staining indistinguishable from nonspecific background staining. This case harbored BRAF‐V600E mutation detected by both Sanger sequencing and ASQ‐PCR. Thus, 4 of 9 cases (44.5%) of the PXA/APXA presented with BRAF‐V600E mutation.

Four PA/PMA (4/60, 6.6%) developed, respectively, in the opto‐chiasmatic region (two cases), the third ventricle and the basal ganglia, expressed BRAF‐V600E in IHC. The immunostaining was cytoplasmic diffuse with moderate to high intensity. Four of 35 supratentorial PA (11.5%) versus none of 23 infratentorial PA were immunopositive.

Statistical analyses did not show any significant difference in BRAF‐V600E expression according to tumor location (cerebellar vs extra‐cerebellar) or age at diagnosis (pediatric vs adult).

### 3.4 Molecular biology

Fifty‐three cases were tested by MB techniques (26 GG, 2 AGG, 14 PA, 4 PXA, 2 APXA, 4 DNT, and one LGGNT). Fifty‐two cases were analyzed by ASQ‐PCR of whom 37 were also tested by Sanger sequencing. One case was tested by Sanger sequencing only. For 6 of 53 cases, DNA was of insufficient quality to perform MB. In two cases, ASQ‐PCR failed but Sanger sequencing could be performed. For the 47 cases that could be assessed, BRAF‐V600E mutation was detected in 41.5% of cases (by Sanger sequencing and/or ASQ‐PCR). Sanger sequencing yielded positive results in 19.5% of cases and ASQ‐PCR in 45.5% of cases. Among the 47 cases assessed, the results were concordant in 41 cases (87.2%) (19 cases with mutation detected by both IHC and MB and 22 cases with BRAF immunonegativity and WT status by MB) and discordant in six cases (Table 3). Five of the six discordant cases were positive in IHC but negative in MB (both ASQ‐PCR and Sanger sequencing were performed in three cases, and only ASQ‐PCR was performed in two cases). Those five cases were GG (four WHO grade I and one WHO grade III). Three displayed an immunostaining restricted to the ganglion cells, and two displayed a more diffuse staining, involving both neuronal and glial components. The last discordant case (PXA, n°133) was equivocal in IHC but harbored BRAF‐V600E mutation detected by both Sanger sequencing and ASQ‐PCR (Table 4). Furthermore, in another eight cases, the mutation was detected by IHC and ASQ‐PCR, but not by Sanger sequencing. In those eight cases, the frequency of the mutated allele, estimated semiquantitatively by ASQ‐PCR, was ≤20%, which corresponds to the detection threshold of the Sanger method (Monzon et al., 2009). In our study, we could not reliably assess IHC sensitivity and specificity because we first selected for sequencing the cases displaying weak or equivocal immunostaining. Because of the limited amount of material in many cases, we first tested the tumors with ambiguous or restricted staining for which IHC could not reliably predict BRAF mutation. This selection bias may have artificially lowered the specificity of the IHC test (specificity of 81.5% and sensitivity of 95% compared to the gold standard (i.e., MB techniques). To determine those values, equivocal cases were considered as negative because a true immunostaining could not be ascertained).

**Table 3:** Comparison between IHC and MB (Sanger sequencing and/or Q‐PCR) results for BRAF‐V600E mutation

	Mutated cases	Wild‐type cases	Total
Positive IHC	19	5	24
Negative IHC	1	22	23
Total	20	27	47

IHC, immunohistochemistry; MB, molecular biology.

**Table 4:** Discordant cases between IHC and Sanger sequencing resolved by ASQ‐PCR

N° case	Location	Diagnosis	IHC	Sanger	ASQ‐PCR	% mutant allele
46	Optic tracts	PA	Moderately positive	WT	V600	5%–10%
70	Cerebral hemisphere	GG	Diffusely positive	WT	V600	5%
72	Cerebellum	GG	Positive ganglion cells	WT	WT	
74	Optic tracts	GG	Rare positive ganglion cells	WT	V600	10%
87	Cerebral hemisphere	GG	Positive ganglion cells	WT	WT	
89	Cerebral hemisphere	GG	Both components positive	WT	V600	20%
96	Cerebral hemisphere	GG	Positive ganglion cells	WT	V600	10%–15%
97	Cerebral hemisphere	GG	Two components moderately positive (5%–10% + cells)	N/A	WT	
100	Cerebellum	GG	Positive ganglion cells	WT	V600	5%
102	Cerebral hemisphere	GG	Both components positive	N/A	WT	
103	Cerebellum	GG	Positive ganglion cells	WT	V600	5%
111	Cerebral hemisphere	GG	Both components positive	WT	V600	10%
125	Cerebral hemisphere	AGG	Moderately positive ganglion cells	WT	WT	
133	Cerebral hemisphere	PXA	Equivocal	V600E	V600	>20%

PA, pilocytic astrocytoma; GG, ganglioglioma; PXA, pleomorphic xantho‐astrocytoma; AGG, anaplastic ganglioglioma.

## 4 Discussion

Histopathologically, PA and mixed GNT are sometimes difficult to distinguish from one another and can be misdiagnosed as diffuse gliomas, especially on biopsy specimens. Definitive diagnosis is based on histopathology, radiological findings, and almost invariably, molecular biomarkers. Because of the sometimes limited access to molecular platforms, the ability to detect a mutant protein by IHC offers a major advantage in daily practice. IHC is fast, inexpensive, and readily available in pathology departments. It can be performed on minute samples as opposed to molecular techniques. Several studies have shown that anti‐BRAF‐V600E IHC had a sensitivity and specificity over 95% in cutaneous melanoma (Capper et al., 2011; Colomba et al., 2013; Long et al., 2013; Ritterhouse & Barletta, 2015). In colon and thyroid cancers, IHC also proved consistent (Bösmüller et al., 2013; He, Zhao, Zhang, & Gong, 2014; Ritterhouse & Barletta, 2015), but its reliability remains to be assessed in large series of primary CNS tumors. We studied BRAF‐V600E expression in 140 glial and GNT. Immunoreactivity with BRAF‐VE1 antibody was detected in 61.5% of GG/GC/AGG, 33% of PXA/APXA, 6.6% of PA/PMA, and in no DNT. Sanger sequencing and/or ASQ‐PCR could be performed in 47 cases. For many specimens, the amount of (FFPE) material available was not sufficient to allow for MB analysis. This fact underlines the key role of IHC in detecting biomarker expression in routine practice. One caveat of our study is that we first selected for sequencing the cases with ambiguous or restricted (few ganglion cells only) staining for which IHC could not reliably predict BRAF mutation. So, no firm conclusion can be drawn from the sensitivity and specificity values in our study. However, the results of BRAF IHC were very good although inferior to those obtained in melanomas (Colomba et al., 2013; Long et al., 2013).

We studied few cases of PXA (nine cases) and DNT (seven cases); this may explain why the frequencies of BRAF‐V600E mutation were lower than those reported in the literature (44.5% vs 66% for PXA/APXA and 0% vs 25% for DNT) (Chappé et al., 2013; Dougherty et al., 2010; Schindler et al., 2011).

As already mentioned, 38.5% of GG/AGG in our series were immunopositive for BRAF‐V600E only in the ganglion cell component. This observation has been reported in the literature but has not been expanded upon (Koelsche et al., 2013). However, this finding leads to discuss the detection threshold of MB techniques. GG harbor a variable proportion of ganglion cells, from a few scattered cells to an authentic gangliocytoma (tumor composed entirely of ganglion cells with no glial tumor component) (Louis et al., 2007). The glial component is most often predominant in GG. In our study, Sanger sequencing did not detect BRAF mutation in 10 cases of GG/AGG that were immunopositive for BRAF. In three of those 10 cases, the immunostaining was detected only in a few ganglion cells but with a significant intensity. In 7 of 10 cases (including two of the three cases with rare immunopositive ganglion cells), the mutation was detected by ASQ‐PCR (Table 4). The discrepancy between the results obtained with the two techniques (Sanger sequencing vs ASQ‐PCR) may be explained by a distinct detection threshold. Sanger sequencing has a detection threshold of 20% (of mutated alleles) compared to 5% for ASQ‐PCR (Monzon et al., 2009). The seven discordant cases had a percentage of mutated allele <20%. The high threshold of Sanger sequencing questions the relevance of this technique in detecting BRAF mutation in GG, which often contain a minority of ganglion cells. IHC appears to be a more robust technique to detect BRAF‐V600E mutation in GNT with a small number of mutant cells. It is of note that anti‐BRAF‐V600E antibody detects only the BRAF‐V600E variant even though cross reactivity with non pV600E mutations has been described (e.g., V600K, V600R) (Ihle et al., 2014). The absence of immunostaining does not rule out another V600 mutation (Ritterhouse & Barletta, 2015). This is also true for ASQ‐PCR, which is designed to detect BRAF‐V600E mutant. Only Sanger sequencing can detect the different variants (but with a lower sensitivity compared to ASQ‐PCR). Of note, we did not identify BRAF mutation other than the V600E variant. Patients with mutation other than BRAF‐V600E are eligible to targeted therapies such as vemurafenib or dabrafenib (Lee et al., 2014; Sosman et al., 2012). Because of the therapeutic implications, the relevance of detecting BRAF mutation is already expanding to other types of CNS tumors, therefore requiring robust detection techniques. It would be interesting to evaluate newer MB techniques in GNT such as next‐generation sequencing or pyrosequencing, which is fast, more sensitive and has a better yield compared to Sanger sequencing (Colomba et al., 2013). Those techniques detect all BRAF mutations but are still expensive and not widely available.

If the presence of BRAF‐V600E mutation is a diagnostic clue to a circumscribed low‐grade glial or glial–neuronal tumor, this mutation is not specific of GNT; it has been reported in rare cases of low‐grade diffuse gliomas in children and in 10% of GB, especially in the epithelioïd variant (which also expresses CD34 more often) (Brastianos et al., 2014; Ichimura et al., 2012; Kleinschmidt‐DeMasters, Aisner, & Foreman, 2015; Kleinschmidt‐DeMasters et al., 2013). The detection of the mutation can still help to distinguish a GG from the cortical infiltration of a diffuse glioma or a GG from an astrocytoma especially in the cerebellum, where PA rarely exhibits BRAF‐V600E mutation (but instead the BRAF‐KIAA1549 fusion in 80% of cases).

As expected, the percentage of BRAF‐V600E mutant PA in our series was low (6.6%), which is comparable to the results obtained in other studies (Chappé et al., 2013; Schindler et al., 2011). It is interesting to note that the four mutant PA in our cohort were all of supratentorial location (basal ganglia, third ventricle, and optic pathways). However, extra‐cerebellar BRAF‐V600E mutant PA remains rare (Roth et al., 2015) leading to wonder whether it is a distinct entity or actually GG whose neuronal component was not sampled.

## 5 Conclusion

The detection of BRAF‐V600E mutation represents a diagnostic aid in glial and GNT. Targeted therapies against BRAF‐V600 mutant proteins have shown promising results. In this context, anti‐BRAF‐V600E IHC plays a key role in clinical practice, especially as it is a fast, inexpensive, and easily accessed technique. In GG, the presence of the mutation in only scattered neuronal cells reduced the sensitivity of Sanger sequencing. However, ASQ‐PCR was able to detect the mutation in few tumor cells. Thus, while waiting for the assessment of newer MB techniques, we should order IHC in addition to ASQ‐PCR, which should be preferred to Sanger sequencing in glioneuronal tumors.

## Conflict of interest

The authors have no conflict of interest to disclose.
